# Association of Warmer Weather and Infectious Complications Following Transrectal Ultrasound-Guided Prostate Biopsy

**DOI:** 10.3390/jpm12030446

**Published:** 2022-03-11

**Authors:** Yu-Chen Chen, Hao-Wei Chen, Shu-Pin Huang, Szu-Huai Lin, Ting-Yin Chu, Ching-Chia Li, Yung-Shun Juan, Wen-Jeng Wu

**Affiliations:** 1 Graduate Institute of Clinical Medicine, College of Medicine, Kaohsiung Medical University, Kaohsiung 80708, Taiwan; jennis7995@hotmail.com (Y.-C.C.); chanhoward21@hotmail.com (H.-W.C.); shpihu73@gmail.com (S.-P.H.); ccli1010@hotmail.com (C.-C.L.); juanuro@gmail.com (Y.-S.J.); 2 Department of Urology, Kaohsiung Medical University Hospital, Kaohsiung Medical University, Kaohsiung 80708, Taiwan; 3 Department of Urology, Kaohsiung Municipal Ta-Tung Hospital, Kaohsiung 80145, Taiwan; 4 Nurse Specialist for Surgery, Specialist Nursing Office, Kaohsiung Medical University Hospital, Kaohsiung 80708, Taiwan; kitty680824@gmail.com; 5 Department of Business Management, Institute of Health Care Management, National Sun Yat-Sen University, Kaohsiung 80424, Taiwan; chu880102@gmail.com

**Keywords:** prostate, transrectal, infection, seasonality, sepsis, temperature, weather

## Abstract

The seasonal and meteorological factors in predicting infections after urological interventions have not been systematically evaluated. This study aimed to determine the seasonality and the effects of the weather on the risk and severity of infectious complications (IC) after a transrectal ultrasound-guided prostate biopsy (TRUS-Bx). Using retrospectively collected data at the tertiary care hospital in Taiwan, we investigated the seasonal and meteorological differences in IC after TRUS-Bx. The IC included urinary tract infection (UTI), sepsis, and a positive culture finding (PCF). The severity was assessed on the basis of the Common Terminology Criteria for Adverse Events grading system. The prevalences of the infectious complications (UTI, sepsis, PCF and grade ≥ 3 IC) were significantly higher in the summer than in the winter. Monthly temperature and average humidity were significant factors for IC. After adjusting the demographic factors, multivariate regression revealed that UTI, sepsis, PCF, and grade ≥ 3 IC increased by 12.1%, 16.2%, 21.3%, and 18.6% for every 1 °C increase in the monthly average temperature, respectively (UTI: *p* = 0.010; sepsis: *p* = 0.046; PCF: *p* = 0.037; grade ≥ 3 IC: *p* = 0.021). In conclusion, the development and severity of IC after TRUS-Bx had significant seasonality. These were dose-dependently associated with warmer weather. Infectious signs after TRUS-Bx should be monitored more closely and actively during warm weather.

## 1. Introduction

Transrectal ultrasound-guided prostate biopsy (TRUS-Bx), a standard procedure used to diagnose prostate cancer, is one of the most commonly performed urologic procedures, with more than one million prostate biopsies performed annually among Medicare beneficiaries [1]. Infection is the most common and serious complication of prostate biopsy. The reported rates of infectious complications range from 0.1% to 7.0%, while the sepsis and hospitalizations rates range from 0.3% to 3.1% [2] and 0.6% to 4.1%, respectively [3]. In addition, there has been a reported increase in the incidence of hospitalizations and fatal sepsis after prostate biopsy [4]. Risk factors for developing infectious complications after TRUS-Bx include a previous history of prostatitis, diabetes, and the development of antimicrobial resistance [5].

However, beyond individual-level risk factors, environmental risk factors for infectious complications may exist. So far, seasonality has been reported for certain bacterial [6,7], viral [8], and even parasitic infections [9]. For the several infections showing a seasonal pattern in their incidence, the variations have been associated with weather changes, such as temperature, humidity, rainfall, or wind [6,7,10]. It is likely that the weather is also related to the infectious complications after TRUS-Bx.

To our knowledge, there have not been any studies investigating the seasonality of infectious complications in patients following TRUS-Bx. We believe that understanding the epidemiological information of post-biopsy infectious complications can help in developing preventive strategies or interventions in clinical care. Therefore, the purpose of this study was to determine the effects of weather on the risk and severity of infectious complications after TRUS-Bx.

## 2. Materials and Methods

### 2.1. Ethics Statement

The study protocol was approved by the institutional review board (IRB) of Kaohsiung Medical University Hospital (IRB No. KMUHIRB-E(I)-20210165). Informed consent was waived by the IRB.

### 2.2. Study Population

Patients who underwent TRUS-Bx between January 2012 and December 2016 in the tertiary medical center were enrolled. The indications for biopsy were high serum prostate-specific antigen (PSA) levels or abnormal findings on digital rectal examination, TRUS, or magnetic resonance imaging that were strongly indicative of prostate cancer. The follow-up after the biopsy had to be at least 1 month for a patient to be eligible. The exclusion criteria were urinary tract infection, acute bacterial prostatitis (National Institutes of Health classification I), chronic bacterial prostatitis (National Institutes of Health classification II), having an indwelling Foley catheter before biopsy, or use of antibiotics for some other reasons within 1 month before the prostate biopsy. Patients who were lost to follow-up, underwent other procedures simultaneously during the biopsy, or underwent other prophylactic antibiotics instead of gentamicin and cefixime were excluded.

### 2.3. Procedures

A standard TRUS-Bx procedure was performed in accordance with the European Association of Urology Guidelines. The urologists performed strict pre- and intraoperative preparations: (1) informed consent from patients was obtained after providing adequate information of the procedure and its potential hazards; (2) prophylactic antibiotics with intramuscular gentamicin and oral cefixime were administered; (3) patients were asked to fast on the day of the biopsy and undergo a rectal-cleaning enema; (4) digital rectal examination was performed before the biopsy; (5) intrarectal iodine perfusion and skin disinfection were performed around the anus; (6) local anesthesia was administered intrarectally. The patients were discharged once they had achieved smooth micturition after the biopsy. All patients returned to the urology outpatient clinic within 2 weeks after the biopsy to receive their pathology report and for us to determine whether any infectious or noninfectious complications had occurred. The patients were also instructed to return to the hospital if they developed any symptoms of infection.

### 2.4. Clinical Characteristics and Climate Data Collection

In this retrospective analysis, we collected the following clinical data from all the enrolled patients: age, body mass index (weight divided by height squared, kg/m^2^), PSA levels, prostate volumes, and comorbidities; the TRUS-Bx pathological reports with Gleason score used to describe the biopsy results. The involvement of any first-year residents was also reviewed.

The seasons were defined as spring (from March to May), summer (from June to August), autumn (from September to November), and winter (from December to February). The climate data of the corresponding months were collected from the Central Weather Bureau, Taiwan. We used the data recorded in the nearest weather station, located 4.5 km away from the hospital. The monthly meteorological data included the average, highest, and lowest temperature (measured in °C), average humidity (recorded in percentage), total rainfall (measured in mm), total rain days (recorded in days), total sunshine hours (recorded in h), average atmospheric pressure (measured in mBar, equivalent to 1 hPa in SI unit), and maximum 10 min wind speed (measured in m/s).

### 2.5. Study Endpoints

The endpoints of the study were the incidence of infectious complications after TRUS-Bx, which included urinary tract infection (UTI), sepsis, and positive culture finding (PCF) within 1 month. Sepsis was defined as the presence of two or more of the following conditions along with urinary tract bacterial infection: temperature >38.0 °C or <36.0 °C, heart rate >90 bpm, respiratory rate >20 breaths/min or respiratory alkalosis, and white blood cell count >12,000 or immature cell form count >10% in proportion [11]. For the patients with bacteriuria, urine culture was collected, while both urine and blood samples were collected for culture and full evaluation among those with febrile UTI or sepsis. The severity of infectious complications was assessed on the basis of the Common Terminology Criteria for Adverse Events (CTCAE): grade 2 = localized infections with oral antibiotics indicated, grade 3 = severe infections with intravenous antibiotic indicated, grade 4 = life-threatening consequences, and grade 5 = death [12]. There is no grade 1 complication defined in the CTCAE grading system among infectious process involving the urinary tract.

### 2.6. Statistical Analysis

The patients’ characteristics are summarized using descriptive statistics. Patients were stratified by the season when the biopsy was performed. The differences of demographic data and infectious complications among the four seasons were analyzed by the chi-square test for categorical variables or by the ANOVA test for continuous variables. To identify the possible risk factors behind seasonality, monthly meteorological variables were univariately analyzed for infectious complications. The multivariable logistic regression analysis was conducted to assess the effects of all clinically relevant demographic and meteorological factors on the infectious complications. Specifically, we used average monthly temperatures in the multivariable logistic regression, while the other meteorological factors were excluded because these were highly correlated with the average monthly temperature. All tests were two-sided, and *p*-values ≤0.05 were considered statistically significant. Statistical analyses were performed using commercial statistical software (Statistical Package for the Social Sciences, version 23.0; SPSS, Inc, Armonk, NY, USA).

## 3. Results

A total of 851 patients were included in this study. Table 1 summarizes the baseline characteristics of all the patients. The mean age was 68.2 years, the mean PSA level was 63.3, and the positive pathological biopsy was 30.3%. One-fifth (21.3%) and one-half (47.6%) of the patients had concomitant diabetes mellitus and hypertension, respectively.

### 3.1. Seasonality Variation of Infectious Complications

Among all TRUS-Bx patients, 57 (6.7%), 22 (2.6%), and 17 (2%) patients developed UTI, sepsis, and PCF; 33 (3.9%) and 25 (2.9%) patients had CTCAE grade 2 and grade ≥ 3 infectious complications, respectively. For the seasonal differences shown in Table 2, significant seasonality was identified among UTI, sepsis, PCF and the severity of infections (*p* = 0.011, 0.028, and 0.002, respectively). The incidences of all the infectious complications (UTI, sepsis, and PCF) were lowest in the winter (3.6%, 0.6%, and 0.6%) and highest in the summer (13%, 5.5%, and 4.5%). When compared with winter, UTI, sepsis, PCF, and grade ≥ 3 infectious complications occurred significantly more often in the summer (*p* < 0.05).

### 3.2. Meteorological Variables behind Seasonality and Univariate Analysis

**Figure 1:**
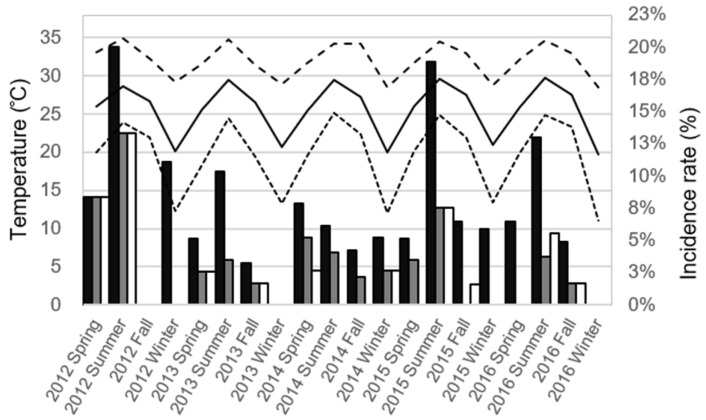
The distribution of seasonal infectious complications rates and temperatures in the continuous 5 year time period in Kaohsiung, Taiwan. The column represents the seasonal rate of infectious complications after a transrectal ultrasound-guided prostate biopsy (black column: urinary tract infection; gray column: sepsis; white column: positive culture finding). The line represents the average monthly temperature during the season (black solid line: average temperature; dense dotted line: lowest temperature; loose dotted line: highest temperature).

Figure 1 demonstrates the distribution of seasonal infectious complications rates and temperatures in the continuous 5 year time period. A univariate analysis was conducted to better understand the association between the infectious complications and climate (Table 3). We found a significant association between UTI and the monthly average temperature (*p* = 0.012), monthly highest temperature (*p* = 0.007), monthly lowest temperature (*p* = 0.019), and average humidity (*p* = 0.007). There was also a significant association between sepsis and the monthly highest temperature (*p* = 0.041). The monthly highest temperature was the only significant factor for PCF (*p* = 0.039). Meanwhile, there were significant associations between grade ≥3 infectious complications and the monthly average temperature (*p* = 0.024), monthly highest temperature (*p* = 0.021), and monthly lowest temperature (*p* = 0.047).

### 3.3. Multivariate Regression Analyses

To identify the potential risk factors for infectious complications, a multivariate logistic regression analysis was performed (Table 4). After adjusting for the patients’ demographic factors, the odds of all infectious complications significantly increased with a higher monthly average temperature. UTI, sepsis, PCF, and grade ≥ 3 infectious complications increased by 12.1%, 16.2%, 21.3%, and 18.6% for each 1 °C increase in the monthly average temperature, respectively (UTI: 95% CI = 1.028–1.223, *p* = 0.01; sepsis: 95% CI = 1.003–1.348, *p* = 0.046; PCF: 95% CI = 1.012–1.454, *p* = 0.037; grade ≥ 3 infectious complications: 95% CI = 1.026–1.370, *p* = 0.021). Furthermore, a higher prostate volume was significantly associated with PCF (adjusted OR, 1.019; 95% CI = 1.001–1.037, *p* = 0.037).

## 4. Discussion

This retrospective cohort study showed that the vast majority of the observed seasonality in the development of infectious complications after TRUS-Bx, including UTI, sepsis, PCF, and the severity of infections, can be explained by the weather, specifically by changes in the temperature. We found that the increased risk for infectious complications was associated with warmer temperatures, and this persisted even after adjusting for patients’ demographic data. To our knowledge, this is the first study to discuss the seasonality of and the meteorological factors affecting infectious complications after TRUS-Bx.

Although TRUS-Bx for the diagnosis of suspected prostate cancer is generally considered safe, infection complications require proper attention. The incidences of UTI following TRUS-Bx are approximately 1% to 17.5%, with an estimated average of 6% [5], which were compatible with the occurrence rate of 6.7% in our cohort. The rate of sepsis in our study was 2.6%, also compatible with the previously reported rates of 0.3% to 4.1% [2,3,5]. In addition, infectious complications negatively affect patients’ health and emotional wellbeing and incur economic costs. Hospitalization for sepsis after TRUS-Bx, estimated to cost 5800 USD per event, is the most serious and fatal complication of TRUS-Bx [13]. In our cohort, 2.4% required hospitalization because of biopsy-related infectious complications, and 0.1% (one patient) died due to severe sepsis and exacerbation of underlying medical conditions. Therefore, it is indeed important to understand the underlying and epidemiological information on post-biopsy infectious complications to develop preventive strategies or interventions in clinical care. Compared to previous studies that focused on patients’ demographic risk factors, such as a previous history of prostatitis, preexisting diabetes, and the development of antimicrobial resistance, for infectious complications [5], we focused on the seasonality and meteorological variables affecting these complications after adjusting for the possible demographic variables. To avoid the possible effect of previous infectious history and to prevent the occurrence of antimicrobial resistance, the patients who had UTI, prostatitis, Foley catheters, and antibiotic use within 1 month before the prostate biopsy were excluded. Due to the fact that the overall resistance to fluoroquinolones is rising [2], we also excluded the patients who underwent fluoroquinolones as prophylaxis. After the strict exclusion and multivariate logistic regression, diabetes was not a risk factor, whereas monthly average temperature was, in our cohort. Although it was reported that diabetes may increase the risk of post-biopsy infectious complications [14], another study demonstrated no significant correlation of diabetes, fasting blood glucose before biopsy, and fever after biopsy [15].

Our analysis revealed that all the infectious complications (UTI, sepsis, and PCF) occurred more significantly in the summer when compared with winter. Meanwhile, the severity of infectious complications had a significant trend of seasonality. Furthermore, we analyzed the data with temperature as the key meteorological variable. After adjusting for the demographic covariables, a dose-dependent relationship between temperature and the development of infectious complications was observed. For every 1 °C increase in the average monthly temperature, the odds of infectious complications increased by 12.1%, 16.2%, 21.3%, and 18.6% for UTI, sepsis, PCF, and grade ≥3 infectious complications, respectively. 

So far, a seasonal trend, especially in summer, has been reported for infections [16], with many being related to warm temperatures [17], and some being related to other meteorological variables [7]. Concerning urology-related infections, UTI and acute pyelonephritis were reported to be associated with warmer weather [18,19]. Sepsis was reported to vary strikingly by season and pathogen [20]. Likewise, the uropathogenic *Escherichia coli* was reported to have a seasonal pattern [21]. The UTI-related hospitalization rate was found to increase by 19% increase in the months when the average temperatures were 27.5–30 °C, compared to when the average temperatures were 5–7.5 °C [22]. In addition, the risk of surgical site infections was reported to be highly seasonal and was associated with warmer weather [23]. However, previous studies concluded that there was a significant difference in morbidity and mortality at the beginning of the inexperienced residents’ training, called the “July effect” [24]. Therefore, in the present study, we adjusted for participation by any first-year residents to limit the “July effect”.

The reasons for the seasonality of the infectious complications after TRUS-Bx are most likely multifactorial. Specifically, it may depend on the warm and wet weather, which may be explained by two biological hypotheses: (1) bacterial flora and (2) urination. First, elevated levels of bacteria, especially Gram-negative bacteria, such as *Escherichia coli* or *Klebsiella*, seem to correlate well with a higher temperature and humidity compared to climates that are cooler and drier, leading to a higher incidence in the summer [25,26,27]. Second, increased temperatures may lead to volume depletion, leading to decreased urinary volume and flow [18]. Decreased urinary flow can hamper the removal of bacteria from the urinary tract, thus increasing the potential for infection [28]. Alternatively, more concentrated urine may increase the likelihood of developing an infection [18]. In addition to the causes of bacterial development in other diseases, which may be transmitted directly by direct contact or acquired from contaminated environments, such as food or water, the bacteria causing infections after TRUS-Bx may have been seeded into the prostate, bladder, and/or bloodstream by the hollow-core biopsy needle traversing the rectum into the prostate and/or bladder during TRUS-Bx [29]. Once the bacteria are introduced into the body, the infection may develop depending mostly on the host defense, bacterial flora, and urination. According to previous studies, urination and bacterial flora seemed to be associated with the climate [18,30], with warm weather possibly altering the host response, which increases the susceptibility of a host to infections [30]. In the present study, after adjusting for the host factors, the presence of bacteria in the culture was also significantly associated with temperature. However, despite not fully knowing the specific mechanism, our results should help increase the clinical suspicion for infectious complications during warmer weather, especially given the possible need for hospitalization to control severe infections. Ultimately, future work will need to determine how warmer temperatures increase the risk for infectious complications, which may help inform preventive approaches. 

In our study, the prostate cancer diagnosis rate was 30.3%, which is slightly lower than the rate reported in the Prostate, Lung, Colorectal, and Ovarian (PLCO) trial (biopsy group, 32.3%) [31]. However, it is higher than the diagnosis rate in the Rotterdam section of the European Randomized Study of Screening for Prostate Cancer (ERSPC) (biopsy group, 12.8%) [32]. The mean ages in these groups (PLCO: 63.7 years, ERSPC: 62.4 years) were lower than the age in our present study. In Taiwan, the prostate cancer diagnosis rate is 36% according to the nationwide database of National Health Insurance in Taiwan [33], while a single-institution study in Taiwan observed a diagnosis rate of 23.6% [34]. The difference in prostate cancer detection rate among TRUS-Bx patients between our study and other studies may be attributed to different populations and individual bias.

Our present study had some limitations. It was a retrospective and single-center study, with a small number of infectious complications due to the relatively low incidence of infectious complications after TRUS-Bx. In addition, the actual room temperature during the TRUS-Bx was not recorded, although, in our hospital, the room temperature is automatically set between 20 and 24 °C throughout the year by a central air-conditioning system. Lastly, actual weather exposure at the individual level and the interaction between different meteorological variables in causing the infectious complications were not evaluated. In our cohort, although the average humidity was significantly correlated to one of the infectious complications (UTI), we investigated the adjusted odds ratio using only the monthly average temperatures, which may have underestimated the relevance of other meteorological factors. Future research should focus on the actual temperature that the patients experience at an individual level and on the interaction between different meteorological variables.

## 5. Conclusions

Despite the limitations of our work, we demonstrated the strong seasonality of infectious complications and the severity of infectious complications after TRUS-Bx. A significant dose-dependent relationship between the average monthly temperature and the risk for infectious complications was identified. Our results may help inform urologists to monitor for infectious signs after TRUS-Bx more closely and actively during warm weather.

## Figures and Tables

**Table 1:** Patients’ baseline characteristic over transrectal ultrasound-guided prostate biopsy.

Characteristics	All Patients
Patients, *n*	851
Age, years, median (range)	67 (33–92)
BMI, kg/m^2^, median (range)	24.7 (15.1–40.2)
PSA level, ng/dL, median (range)	10.9 (0.7–5342)
Prostate volume, mL, median (range)	44.9 (10.6–182.3)
PSA density, ng/mL^2^, median (range) ^a^	0.27 (0.02–90.3)
Biopsy pathological result, *n* (%)	
No malignancy	593 (69.7%)
ISUP grade 1	69 (8.1%)
ISUP grade 2	22 (2.6%)
ISUP grade 3	25 (2.9%)
ISUP grade 4	66 (7.8%)
ISUP grade 5	76 (8.9%)
Diabetes, *n* (%)	181 (21.3%)
Hypertension, *n* (%)	405 (47.6%)

^a^ PSA density was calculated as total PSA (ng/mL) divided by prostate volume (mL). Abbreviations: BMI = body mass index; PSA = prostate-specific antigen; ISUP = International Society of Urological Pathology.

**Table 2:** Baseline characteristics and incidence of infectious complication after TRUS-Bx per season and comparisons among different seasons.

	Subgroup				
	Spring	Summer	Fall	Winter	*p*-Value
	*N* = 210	*N* = 200	*N* = 244	*N* = 197	
**Baseline characteristics**					
Age, years, median	67	66	69	68	0.675
BMI, kg/m^2^, median	24.8	24.7	24.4	24.7	0.209
PSA level, ng/dL, median	10.9	11.0	11.0	10.2	0.852
Prostate volume, mL, median	46.4	47.0	42.0	42.8	0.537
PSA density, ng/mL^2^, median ^b^	0.26	0.27	0.27	0.26	0.744
Positive biopsy result, *n* (%)	55 (26.2%)	63 (31.5%)	84 (34.4%)	56 (28.4%)	0.275
Diabetes, *n* (%)	44 (21.0%)	42 (21.0%)	59 (24.2%)	36 (18.3%)	0.527
Hypertension, *n* (%)	93 (44.3%)	99 (49.5%)	116 (47.5%)	97 (49.2%)	0.726
**Infectious complications**					
Urinary tract infection, *n* (%)	13 (6.1%)	26 (13%) ^a^	11 (4.5%)	7 (3.6%)	<0.001
Sepsis, *n* (%)	6 (2.9%)	11 (5.5%) ^a^	4 (1.6%)	1 (0.6%)	0.011
Positive culture findings, *n* (%)	3 (1.4%)	9 (4.5%) ^a^	4 (1.6%)	1(0.6%)	0.028
**Infectious Severity, CTCAE grade**					0.002
Grade 2, *n* (%) Grade ≥ 3, *n* (%)	7 (3.3%)	14 (7.0%)	6 (2.5%)	6 (3.0%)	
	6 (2.9%)	13 (6.6%) ^a^	4 (1.6%)	2 (1.0%)	

^a^ Versus winter (*p* < 0.05). ^b^ PSA density was calculated as total PSA (ng/mL) divided by prostate volume (mL). Abbreviations: TRUS-Bx = transrectal ultrasound-guided prostate biopsy; CTCAE = Common Terminology Criteria for Adverse Events, version 5.

**Table 3:** Univariable analysis of monthly meteorological parameters for infectious complications.

Variables	UTI		Sepsis		PCF		CTCAE Grade ≥ 3	
	OR (95% CI)	*p*-value	OR (95% CI)	*p*-value	OR (95% CI)	*p*-value	OR (95% CI)	*p*-value
Average temperature	1.117 (1.025–1.218)	0.012 ^a^	1.156 (0.999–1.339)	0.052	1.194 (0.999–1.427)	0.051	1.180 (1.022–1.362)	0.024 ^a^
Highest temperature	1.180 (1.046–1.331)	0.007 ^a^	1.224 (1.008–1.486)	0.041 ^a^	1.266 (1.012–1.585)	0.039 ^a^	1.243 (1.033–1.493)	0.021 ^a^
Lowest temperature	1.074 (1.012–1.140)	0.019 ^a^	1.082 (0.982–1.192)	0.110	1.099 (0.980–1.233)	0.106	1.101 (1.001–1.211)	0.047 ^a^
Average humidity	1.100 (1.026–1.178)	0.007 ^a^	1.076 (0.968–1.197)	0.176	1.136 (0.999–1.291)	0.052	1.097 (0.991–1.215)	0.074
Precipitation	1.001 (1.000–1.002)	0.111	1.001 (0.999–1.002)	0.406	1.001 (0.999–1.003)	0.211	1.001 (0.999–1.002)	0.245
Sun hours	1.005 (0.998–1.011)	0.171	1.002 (0.992–1.012)	0.685	0.996 (0.985–1.008)	0.552	1.001 (0.992–1.011)	0.785
10 min wind speed	1.061 (0.980–1.150)	0.144	1.038 (0.911–1.183)	0.578	1.091 (0.955–1.246)	0.201	1.054 (0.936–1.186)	0.389

^a^*p* < 0.05. Abbreviations: UTI = urinary tract infection; PCF = positive culture finding; CTCAE = Common Terminology Criteria for Adverse Events, version 5; OR = odds ratio; CI = confidence interval.

**Table 4:** Multiple logistic regression of potential factors on the infectious complications after TRUS-Bx.

Variables	UTI		Sepsis		PCF		CTCAE Grade ≥ 3	
	Adjusted OR (95% CI)	*p*-value	Adjusted OR (95% CI)	*p*-value	Adjusted OR (95% CI)	*p*-value	Adjusted OR (95% CI)	*p*-value
Age	1.000 (0.967–1.034)	0.987	0.996 (0.945–1.049)	0.867	1.012 (0.953–1.076)	0.691	1.008 (0.960–1.059)	0.740
BMI	1.010 (0.927–1.100)	0.827	0.916 (0.790–1.062)	0.243	0.918 (0.778–1.083)	0.309	0.922 (0.803–1.058)	0.245
PSA level	1.000 (1.000–1.001)	0.242	1.001 (1.000–1.001)	0.140	1.000 (0.999–1.001)	0.939	1.000 (1.000–1.001)	0.172
Prostate volume	1.008 (0.997–1.019)	1.154	1.010 (0.993–1.028)	0.247	1.019 (1.001–1.037)	0.037 ^a^	1.009 (0.992–1.026)	0.288
Biopsy pathological result								
No malignancy	Ref.		Ref.		Ref.		Ref.	
Malignancy	0.819 (0.425–1.577)	0.550	1.212 (0.450–3.260)	0.704	1.359 (0.448–4.124)	0.588	0.881 (0.348–2.229)	0.789
Diabetes	0.910 (0.457–1.812)	0.789	0.543 (0.147–2.009)	0.360	0.830 (0.225–3.060)	0.780	1.555 (0.495–4.888)	0.450
Hypertension	1.248 (0.703–2.216)	0.449	0.923 (0.369–2.308)	0.864	0.766 (0.270–2.177)	0.617	1.025 (0.435–2.416)	0.955
The involvement of first year of residency	0.864 (0.499–1.496)	0.601	1.100 (0.454–2.667)	0.833	0.803 (0.302–2.139)	0.661	0.893 (0.388–2.056)	0.790
Monthly average temperature	1.121 (1.028–1.223)	0.010 ^a^	1.162 (1.003–1.348)	0.046 ^a^	1.213 (1.012–1.454)	0.037 ^a^	1.186 (1.026–1.370)	0.021 ^a^

^a^*p* < 0.05. Abbreviations: TRUS-Bx = transrectal ultrasound-guided prostate biopsy; UTI = urinary tract infection; PCF = positive culture finding; CTCAE = Common Terminology Criteria for Adverse Events, version 5, OR = odds ratio, CI = confidence interval, BMI = body mass index, PSA = prostate-specific antigen.

## Data Availability

Deidentified individual participant data that underlie the results reported in this article will be available on request. Applicants must provide (1) a methodologically sound approach to achieve scientific aims, and (2) formal documents of Ethics Committee approval of applicant’s institution. Data will be made available pending authorization of the Institutional Review Board of Kaohsiung Medical University Hospital that will review the applicant’s request and after signing an appropriate data sharing agreement. Proposals should be directed to wejewu@kmu.edu.tw. Data will be available following publication.
